# Robotic Rectus Abdominis Myoperitoneal Flap for Posterior Vaginal Wall Reconstruction: Experience at a Single Institution

**DOI:** 10.3390/jcm14010292

**Published:** 2025-01-06

**Authors:** Noama Iftekhar, Kathryn Cataldo, Seungwon Jong Seo, Brett Allen, Casey Giles, Matthew William Kelecy, Joshua MacDavid, Richard C. Baynosa

**Affiliations:** 1 Department of General Surgery, University of Nevada Las Vegas School of Medicine, Las Vegas, NV 89102, USA; seungwon.jong@unlv.edu (S.J.S.); brett.allen@unlv.edu (B.A.); matthew.kelecy@unlv.edu (M.W.K.); 2 Department of Plastic Surgery, University of Nevada Las Vegas School of Medicine, Las Vegas, NV 89102, USA; kathryn.cataldo@unlv.edu (K.C.); casey.giles@unlv.edu (C.G.); joshua.macdavid@unlv.edu (J.M.); richard.baynosa@unlv.edu (R.C.B.)

**Keywords:** robotic rectus abdominis flap, robotic surgery, plastic surgery, reconstructive surgery

## Abstract

**Background:** The adoption of robotic surgery has been widespread and increasing amongst gynecologic surgeons given the ability to decrease morbidity. It is important that plastic surgeons adjust their reconstructive algorithm to ascertain the benefits of robotic-assisted surgery. Herein we report our outcomes of robotic-assisted rectus abdominis muscle reconstruction of the posterior vaginal wall along with a current literature review on robotic-assisted reconstructive pelvic surgery. **Methods**: An IRB-approved retrospective review was completed of all patients who underwent robotic pelvic reconstruction between 2016 and 2024 at a single institution. Patients who underwent posterior vaginal wall reconstruction utilizing a robotic-assisted rectus abdominis muscle (RRAM) were selected for final analysis. **Results**: Thirty-two patients were identified who underwent pelvic reconstruction using robotic surgical techniques. Five (mean age = 56.2, 32–72; mean BMI = 30.0, 24–39.9) underwent posterior vaginal wall reconstruction with an RRAM flap. Two patients (40%) had minor wound complications, and one patient (20%) had vaginal stenosis eight years after operation. None had major complications requiring a return to the OR or hospital admission. All patients went on to achieve successful healing. **Conclusions**: In the literature, robotic-assisted surgery has shown significant advantages, including reduced morbidity with decreased intra-operative blood loss, reduced pain, faster recovery, and shorter hospital stays. The RRAM flap for pelvic reconstruction is well tolerated in patients despite comorbidities and preserves the minimally invasive benefits of extirpative surgery. As the technology becomes more widely incorporated, it is important for plastic surgeons to integrate robotic surgical techniques into their practice.

## 1. Introduction

The National Aeronautics and Space Administration (NASA) and the United States military initially invested in robotic-assisted techniques in hopes of creating a “telepresence” surgery where the surgeon is not immediately present at the bedside [1,2]. However, due to technological constraints, this effort was transferred to Intuitive Surgical for their DaVinci platform. A major focus in modern surgery has been to reduce the invasiveness of operative approaches, and robotics plays a huge role in ushering in this new era. Robotic-assisted techniques have benefited both the operating surgeons and patients by enhancing dexterity and precision with motion scaling for the operating surgeon [1,3,4,5,6]. Multiple studies have found reduced morbidity for patients with decreased intra-operative blood loss, shorter hospital stays, reduced pain, and faster recovery. Furthermore, the three-dimensional visualization and magnified fields have expanded the feasibility of complex procedures.

The Food and Drug Administration approved the use of the DaVinci operating system for gynecologic surgery in 2005 [7]. Since then, the use of robotic-assisted laparoscopic surgery has become increasingly popular. Between 2007 and 2010 alone, there was an increase from 0.5% of hysterectomies being completed robotically to nearly 10% [8]. Robotic-assisted laparoscopic surgery is solidified in the treatment of benign gynecologic conditions [8,9,10,11]. Myomectomies and hysterectomies have demonstrated decreased blood loss when compared to vaginal or open techniques. The use of the DaVinci system is particularly useful in patients who are obese, have prior adhesive disease, and those with severe endometriosis. Robotic-assisted laparoscopic surgery has decreased conversion to laparotomy when compared to traditional laparoscopic surgery. Sacrocolpopexies and sacrohysteropexies benefit from enhanced precision and wrist function with robotic-assisted techniques compared to laparoscopic techniques [12,13]. Suturing time has been noted to be shorter, and the management of mesh and suture complications from sling procedures has been improved with robot use. Additionally, robotic surgery has been shown to reduce conversion to open incisions in endometrial and cervical cancers [14,15,16]. Furthermore, there have been fewer reported complications and bladder injuries with the use of these robotic techniques. The ability to perform surgery in multiple quadrants is particularly beneficial for complex gynecologic cases.

Our institution, the University of Nevada Las Vegas, has previously published one of the largest studies involving the robotic rectus abdominis flap for perineal repair [17]. We demonstrated that minimally invasive repair could be used even with a large dead space volume and in cases requiring vaginal repair. A non-significant increase was found in minor complications (wound dehiscence) in patients with open flaps. We found no significant differences in major complications in surgical time and length of stay between RRAM and open techniques in this initial study, which included patients until 2021. We found that avoidance of the skin paddle and subcutaneous fat bulk associated with the vertical rectus abdominis myocutaneous flap reduced the risk of vaginal stenosis in the RRAM. The peritoneum on the deep surface of the harvested RRAM flap also matched the native tissue in terms of strength and flexibility for vaginectomy defects.

In this study, we hope to add to the literature regarding our experience and outcomes of using RRAM harvest for vaginal reconstruction.

## 2. Methods

We completed an IRB-approved retrospective review of all robotic rectus abdominis (RRAM) flaps harvested for perineal reconstruction by a single surgeon at a single institution using the da Vinci Surgical System between 2014 and 2024. Thirty-two patients were identified (mean age = 59.9 years; number of females = 12). From this, we then identified patients who underwent posterior vaginal wall reconstruction using RRAM techniques for qualitative analysis and descriptive statistics.

## 3. Results

### 3.1. Case 1

The patient presented with biopsy-proven invasive rectal adenocarcinoma. She had preoperative chemoradiation, low anterior resection, and postoperative adjuvant chemotherapy. She developed a rectovaginal fistula and required a diverting colostomy a year later. The patient then had a primary vaginal fistula repair with a coloanal flap, as well as another low anterior resection with an end colostomy. She was found to have a recurrence in the rectum. As a result, she had an abdominoperineal resection with vaginal reconstruction using the robotic rectus (RRAM) flap. She was noted to have significant scar tissue due to her prior surgeries and radiation. She required a more lateral port placement due to her significant scar tissue to improve visualization. The muscle was taken down superiorly to inferiorly, and the rectus sheath was taken down to the level of the pedicle. The posterior rectus sheath and peritoneum were secured to the edges of the vaginal wall in an interrupted fashion. She was then re-inspected with a speculum and good approximation and closure were noted. While she had no initial wound complications, she developed an adenocarcinoma recurrence at her vaginal wall and required multiple rounds of chemotherapy and additional vaginal brachytherapy. She was noted to have vaginal stenosis eight years after the RRAM procedure.

### 3.2. Case 2

The patient presented with Bartholin gland carcinoma that extended through the posterior vaginal wall, rectum, and anus. She had an abdominoperineal resection completed as a joint case with colorectal surgery and gynecology oncology. An RRAM flap was utilized by plastic surgery for reconstruction of the posterior vaginal wall, as well as for filling of the new pelvic defect, rectal vault, and closure of the perineum. Additional ports were placed in the left lateral abdomen to identify the deep inferior epigastric artery pedicle in the right lower quadrant. The falciform ligament was also taken down in this process to obtain additional RRAM flap length superior to the costal margin. The total overall length of closure was 16 cm. The patient performed well postoperatively with some superficial perineal dehiscence that was initially treated with packing changes for one month and hypergranulation tissue that was treated with gentian violet.

### 3.3. Case 3

This patient developed a rectovaginal fistula after prior low anterior resection for rectal cancer and completion of chemotherapy and radiation. The patient had developed soft tissue damage to the area secondary to radiation. An RRAM flap was performed after the initial lysis of adhesions and repair of rectovaginal fistula with closure of the rectal stump by colorectal surgery. Two separate ports were placed, and the pedicle was identified and isolated for a total area of 7 × 8 cm^2^ of the myoperitoneal flap to buttress the repair with a well-vascularized flap in previously irradiated tissue. She had a reinforcement with a biologic mesh at the site of anterior repair. The patient tolerated the repair well, without any wound complications. Her hospital course was prolonged due to a left ureter injury during the initial lysis of adhesions, for which she required a Foley catheter and nephrostomy tube placement.

### 3.4. Case 4

**Figure 1:**
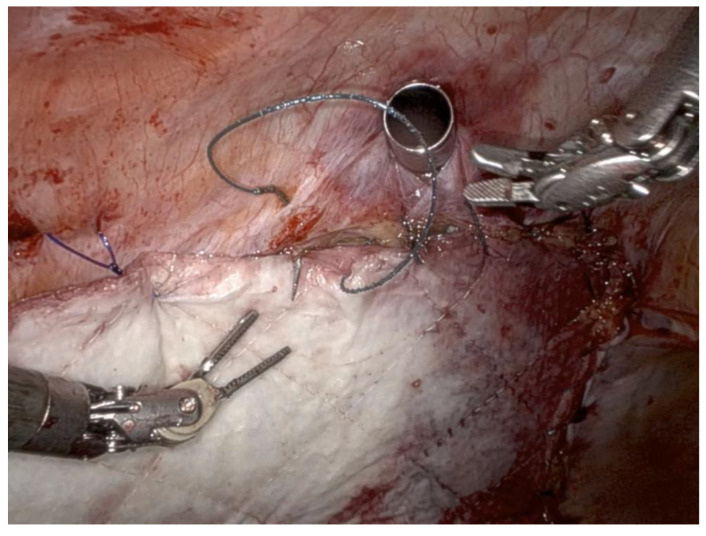
Reinforcement of the repair with a strattice mesh using a barbed suture.

Plastic surgery was consulted for pelvic floor reconstruction for a patient with recurrent adenocarcinoma of the rectosigmoid junction. She had already undergone low anterior resection with loop ileostomy and subsequent ostomy reversal after completion of her chemoradiation. She subsequently underwent robot-assisted laparoscopic abdominoperineal resection followed by reconstruction of the perineum and posterior vaginal wall with right RRAM flap harvest and abdominal wall reinforcement with a strattice mesh. Figure 1 demonstrates reinforcement with the biological mesh. The postoperative course was uncomplicated.

### 3.5. Case 5

The patient presented for combined surgery with colorectal and plastic surgery after a known history of squamous cell carcinoma of the anal canal that failed chemotherapy and radiation, as well as a low anterior resection. She experienced a recurrence invading the rectum and posterior vaginal wall and subsequently underwent robot-assisted laparoscopic abdominoperineal resection followed by reconstruction with a robotic rectus abdominis flap. Follow-up imaging was negative for intra-abdominal abscess or recurrent disease, and the remainder of her postoperative course was uncomplicated.

## 4. Discussion

Dead space refers to a cavity secondary to tissue loss from trauma, infection, or resections. Dead space poses a risk for morbid complications, largely hernia development and fluid accumulation, which can act as a nidus for infection and ultimately deep space abscess [18,19]. Pelvic exenteration for advanced/recurrent gynecologic cancers creates large defects for the purpose of achieving negative oncologic margins. Reconstructive surgery is used concomitantly with many complex urogynecological surgeries to reinstate normal anatomy and reduce the dead space. Reconstruction can also address pathologies like pelvic organ prolapse, vaginal agenesis, and fistulas.

Flap surgery is fundamental to reconstruction after these gynecologic procedures and can even restore the function of pelvic structures including the canal, perineum, and vulva [20,21,22,23]. Flaps involve tissue transfer from one part of the body to another to remedy loss of skin, fat, muscle, or bone. While these relocations can be from areas adjacent to the defects, pedicled or free flaps can be transferred over a large distance and are widely employed by plastic and reconstructive surgeons attempting to address large pelvic defects [22]. Perforator flaps rely on vessels that perforate through deeper tissues of the underlying muscle or fascia to reach subcutaneous skin and tissue [24]. Pedicled flaps are used to reconstruct tissue defects without disrupting the native vessel. Free flaps are removed from their native site and require anastomosis of the vessels at the destination. Gracilis, omental, rectus abdominis, gluteus maximus, and anterolateral thigh flaps are commonly selected for wound coverage and restoration of tissue bulk in the pelvis [22]. As gynecologic surgeons shift towards minimally invasive approaches to reduce patient morbidity, there is a rising need for flap harvest to proceed in a similar fashion. Incorporation of the robot into gynecologic/pelvic reconstruction provides an option to streamline the total procedure, reduce donor-site morbidity, and optimize minimally invasive principles.

**Figure 2:**
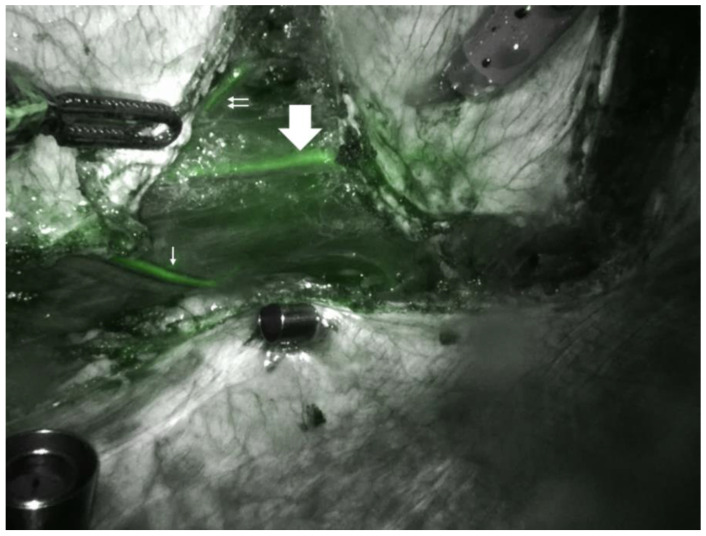
Intra-operative photo of indocyanine green used for the identification of the deep inferior epigastric pedicle for the flap. White arrow is the vessel (highlighted pedicle).

The rectus abdominis flap is a workhorse in abdominal/pelvic reconstruction. It effectively eliminates dead space which is a core principle in pelvic reconstruction. The traditional rectus abdominis flap (VRAM) uses an open approach with a vertically oriented skin paddle and incisions to access the rectus abdominis and elevate the flap [17,25,26,27,28,29,30,31,32,33,34]. It is particularly versatile due to its dual blood supply from the superior and inferior epigastric arteries [17,25]. Figure 2 demonstrates an intra-abdominal view of the inferior epigastric pedicle during a rectus abdominis harvest. However, the morbidity associated with these open approaches can mitigate the benefits achieved by minimally invasive approaches used in large pelvic resections.

As colorectal surgeons and gynecology oncologists more widely utilize a minimally invasive approach, the defects produced are smaller than with traditional laparotomy approaches. Many times, they are able to isolate the tumor as it abuts the vaginal wall due to clearer visualization. Previously, with wider resections, the incorporation of the skin paddle with VRAM techniques was more useful. However, the vaginal wall is hairless and has histological differences from the skin paddle. Drs. Wu and Song from the University of Chicago described five patients between 2003 and 2005 who underwent a rectus abdominis myoperitoneal flap for vaginal wall reconstruction [35]. Their case series demonstrated the viability of these flaps as well as the incorporation of the peritoneum into vaginal mucosa by two months postoperatively. The RRAM incorporates harvest of the peritoneum, which has been shown to have additional benefits in terms of flap healing in rat and swine models [36]. The myoperitoneal flap was shown to have uroepithelium metaplasia by two weeks [3]. Another study utilized the open rectus abdominis myoperitoneal flap for vaginal reconstruction, demonstrating indifferentiable distinctions between vaginal mucosa and the peritoneum histologically [35,37].

The robotic intraperitoneal harvest of the rectus abdominis has been gaining traction among surgeons for pelvic reconstruction over the past two decades for a multitude of reasons beyond preserving the minimally invasive benefits of the extirpative surgery—the decreased risk of incision complications and hernia, improved replication of gynecologic structures, and donor-site aesthetics [24,25,26,27,28,29,30,31,32,33,34]. The RRAM includes a three-port surgical technique and Airseal (Conmed, Largo, FL, USA) for adequate pneumoperitoneum. The posterior fascia is incised using electrocautery after tracing the pedicle (vessels at the origin at the external iliac artery and vein) through the peritoneum. The mesh is then secured to the posterior fascial defect with a barbed suture. The posterior rectus fascia with the underlying peritoneum is then used to create a pelvic floor while off-console. A more comprehensive description of our RRAM harvest technique has been previously published [17].

Our institution has completed thirty-two RRAM procedures for pelvic reconstruction since 2014. Five of these patients (mean age = 56.2, 32–72; mean BMI = 30.0, 24–39.9) underwent posterior vaginal wall reconstruction with an RRAM flap as previously discussed. A summative chart of our patients is detailed in Table 1. All of our patients had received preoperative neoadjuvant chemoradiation. None had major complications requiring a return to the operating room or hospital admission. All patients went on to achieve successful healing.

One patient had vaginal stenosis eight years after the operation. It is important to note that this patient required adjuvant brachytherapy and had a recurrence directly to her vaginal wall. While radiation is particularly effective in cancer cells, due to the induction of apoptosis and G1/S phase arrest in rapidly dividing cells, it also induces significant damage to healthy surrounding tissue [38,39]. Radiation damages blood vessels and halts angiogenesis. This ultimately compromises tissue integrity. Radiation-induced vaginal stenosis is an established side effect of direct pelvic radiotherapy. This is due to excess scar tissue development secondary to collagen deposits in the vaginal connective tissue causing fibrosis and hyalinization [40].

Two of our patients (⅖) had minor wound complications. The literature reports wound complications to be between 25 and 79% for VRAM flaps in abdominoperineal reconstruction. Our prior study, which had incorporated sixteen RRAM flaps, found a minor wound complication rate of 31%, compared to our VRAM group with a complication rate of 55%. These numbers can also be accounted for by the damage associated with radiation, as it is reported that 92% of patients requiring VRAM flaps also require radiation for treatment purposes.

Our data set presented in this paper is small (*n* = 5) and, as a result, requires a more qualitative analysis. It is difficult to extrapolate data based on a small number of patients, but we have been able to demonstrate the feasibility of plastic surgeons in performing the harvest, repair, and flap inset.

A case series completed by researchers at the Mayo Clinic Arizona examined six patients who received rectus abdominis harvest for pelvic floor reconstruction [32]. Patients had a wide variety of pathologies including complex pelvic organ prolapse, vesicovaginal fistula, gynecologic cancer requiring exenteration, vaginectomy, vulvectomy, and abdominoperineal resection. Despite significant comorbidities and poor tissue integrity associated with this group, the pelvic wound demonstrated adequate healing in all six patients. Another case from the Mayo Clinic discussed a woman with recurrent vesicovaginal fistula after uterine cancer that was unsuccessfully treated with conservative and robotic-converted-to-open repair [41]. The use of a robotic rectus abdominis flap, however, when interposed between the vagina and bladder provided successful closure of this fistula.

Other more recent developments include the use of robotically harvested peritoneal flaps to create the apex of the neo-vagina in gender-affirming vaginoplasties. Researchers from New York University reported an additional 5 cm of depth in the vaginal canal among their patient population without any reported complications [42].

## 5. Conclusions

Robotic surgery poses several major advantages. The robotic arms assist in intricate dissection, eliminate operator tremors, and allow for dissection deep in the pelvis without typical ergonomic limitations [17,25,26,27,28,29,30,31,32,33,34]. Furthermore, the camera system on the DaVinci platform allows for incredible visualization and magnification of the field, which would not otherwise be feasible in an open or purely laparoscopic case. Enhanced visualization with the robotic platform is one of the biggest assets and is particularly helpful when operating in the pelvis. Increased instrument articulation is invaluable in tight spaces. Additionally, there are reports of a faster recovery due to smaller incisions and reduced tissue trauma. The smaller incisions often correspond to a better aesthetic outcome.

Robotic surgery is not without limitations. There is a steep initial learning curve for the surgeon and staff which comes with learning how to dock the robot, becoming familiar with the console, and other infrastructure-related learning points [6]. As a result, the initial operative duration may appear longer as the surgeon initially gains familiarity with the system. The ease of the dissection also improves with more experience. Dynamic and non-standardized robot credentialing requirements among hospitals pose another barrier to surgeon access [1]. Additionally, from a technical standpoint, there is a lack of haptic feedback that is present with laparoscopy. Robotic surgery is often more costly due to specialized equipment and initially longer operating room times. However, several other long-term studies in other minimally invasive fields have demonstrated that this cost is diminished when accounting for the decrease in postoperative hospital stay [43]. There are limited studies in the literature regarding the role of robotics in reconstruction specifically. Much of the literature available is presented in case format, making it difficult to fully extrapolate based on the presented data.

Robotics are being applied beyond existing systems with new innovations focused on incorporating robotics into microsurgery and pairing fine motor movements. The fifth-generation DaVinci platform incorporates haptic feedback, which overcomes many of the prior criticisms of robotic surgery [44]. The Medtronic Hugo robotic surgery system brings a new intracorporeal robotic system to the market to perhaps be a real competitor to the current Intuitive DaVinci dominance in this space [45]. The Musa-3 platform, directed towards microsurgeons, has vast potential for intracorporeal vascular anastomoses [46]. There are continued adaptations to improve ergonomics, which can increase the career span of a surgeon.

In conclusion, robotic-assisted surgical procedures have been shown to be not only feasible but also safe as a plastic and reconstructive surgery option after robotic pelvic surgery. The current literature demonstrates that robotic surgery techniques such as the robotic rectus abdominis muscle (RRAM) flap have at least an equivalency of outcomes to traditional open techniques. There is also the potential for superiority over open techniques once the learning curve is overcome. Further research is necessary to demonstrate these improvements and to demonstrate not only the perceived but also the data-driven benefits of utilizing robotic techniques in plastic and reconstructive surgery.

## Figures and Tables

**Table 1:** Demographic overview of patients receiving robotic rectus abdominis flap for vaginal wall reconstruction.

Patient	Age	Pathology	Concomitant Operation	BMI kg/m^2^	Complication
Case 1	32	Rectovaginal fistula, rectal cancer	Abdominoperineal resection	24.0	Vaginal stenosis
Case 2	33	Bartholin gland carcinoma	Abdominoperineal resection	39.9	Minor wound complications amenable to topical treatment
Case 3	66	Rectovaginal fistula secondary to rectal cancer	Takedown of rectovaginal fistula	26	No reported complications from flap (ureter injury from initial fistula takedown)
Case 4	72	Adenocarcinoma of rectosigmoid junction	Abdominoperineal resection	20.9	No reported complications
Case 5	78	Squamous cell cancer of the anus	Abdominoperineal resection	39.9	No reported complications

## Data Availability

The original contributions presented in this study are included in the article. Further inquiries can be directed to the corresponding author.
